# Into the Unknown: Uncertainty and Procrastination in Students From a Life History Perspective

**DOI:** 10.3389/fpsyg.2021.717380

**Published:** 2021-08-18

**Authors:** Amy J. Lim, Sasha Javadpour

**Affiliations:** Discipline of Psychology, College of Science, Health, Engineering and Education, Murdoch University Singapore, Singapore, Singapore

**Keywords:** COVID-19, higher education, uncertainty, procrastination, life history strategy

## Abstract

While existing findings have established an increase in procrastination among students in higher education during COVID-19, they do not elucidate how COVID-19 has effected an increase in procrastination. Drawing upon previous findings and employing a life history framework, this paper proposed that the increase in procrastination may be attributed to the heightened levels of uncertainty in the pandemic. Additionally, this paper examined life history strategy as the psychological mechanism underlying the relation between uncertainty and procrastination. By collecting data across two school semesters in a university (*N* = 253), we found that uncertainty and procrastination did not differ between the semester where changes were abruptly imposed to stem the spread of COVID-19 and the following semester. Our findings also revealed that uncertainty predicted procrastination, and that life history strategy mediated the relation between uncertainty and procrastination. Specifically, uncertainty was associated with a faster life history strategy, which was positively associated with procrastination. By shedding light on the psychology behind the effect of uncertainty on procrastination, the findings of this paper hold important implications for the design of procrastination interventions for the uncertain climate during the pandemic and “the new normal” post COVID-19.

## Introduction

COVID-19 has unequivocally disrupted the academic life of students (Organization for Economic Cooperation and Development, 2020; Chaturvedi et al., 2021). Studies examining the impact of COVID-19 reported decreases in study hours and increases in academic procrastination among students in higher education (Aucejo et al., 2020; Biricik and Sivrikaya, 2020; Jia et al., 2020). Additionally, the nature of online learning from home further encourages procrastination as students not only need to exert higher levels of self-control to overcome isolated learning and the challenges of online learning (Drumm and Jong, 2020; Rasheed et al., 2020; Hong et al., 2021), they must also resist distractions present at home (e.g., television and social media, Meier et al., 2016; Pan, 2020). Taken together, these studies evidenced that procrastination was at higher than average levels during the pandemic.

With the adverse effects procrastination has on academic performance and the effectiveness of online learning during COVID-19 (Kim and Seo, 2015; Hong et al., 2021), attention has been called to manage and reduce the increased procrastination observed in the pandemic (Arifiana et al., 2020). Yet, existing studies do not elucidate how COVID-19 has effected an increase in procrastination. Without understanding how COVID-19 has introduced a preponderance of procrastination, efforts to curb procrastination would be ineffective. Drawing upon previous findings and employing a life history framework (Griskevicius et al., 2013; Del Giudice et al., 2015; Chen and Qu, 2017), this paper proposed that the increase in procrastination may be attributed to the heightened levels of uncertainty in the pandemic.

### Uncertainty in the Air

Uncertainty was at an alarmingly elevated level when the COVID-19 pandemic was declared as a global health emergency (Ahir et al., 2019; Rettie and Daniels, 2020). The looming state of uncertainty was also undoubtedly experienced by students in higher education. Students faced concerns of becoming sick and the possibility that they may lack financial resources to complete their studies (Biricik and Sivrikaya, 2020; Jenei et al., 2020). Additionally, with the closure of schools and the abrupt shift from physical to online learning, students faced uncertain futures of their academic and professional careers (Jenei et al., 2020). A survey of 1,500 university students revealed that students withdrew from classes, changed their majors, delayed their graduation, and expressed less confidence in finding a job by graduation as a result of COVID-19 (Aucejo et al., 2020).

Procrastination, defined as the deliberate delay of a planned course of action, was found to be associated with negative emotional states such as anxiety and stress (Steel, 2007; Hen and Goroshit, 2014). Given that the uncertain climate introduced by the pandemic has resulted in several negative psychological consequences for students, including anxiety and frustration, it is likely that increased procrastination stemmed from the uncertainty experienced by students during the pandemic (Biricik and Sivrikaya, 2020; Brooks et al., 2020; Jenei et al., 2020; Wang and Zhao, 2020; Rahimi and Vallerand, 2021). Existing findings provide initial support for this prediction as they demonstrated that environmental unpredictability was positively related to procrastination (Chen and Qu, 2017).

To better effect strategies and interventions to reduce procrastination among students in higher education, it is important to also understand the underlying psychological mechanism through which procrastination occurs. Without understanding the psychology behind the effect of uncertainty on procrastination, preventing procrastination in a pandemic would be ineffective especially since an uncertain climate is likely to persist as people figure out “the new normal” post COVID-19. As such, this paper employed the life history theory in examining the underlying psychological mechanism behind uncertainty and procrastination.

### Life History Theory and Procrastination

The life history theory posits that organisms allocate limited resources, energy, and time based on environmental constrains (Del Giudice et al., 2015). Life history strategies exist on a slow-fast continuum and are determined by the most optimal allocation of resources, energy, and time between somatic and reproductive effort in response to environmental conditions (Griskevicius et al., 2013; Del Giudice et al., 2015). A slower life history strategy evolved in favorable and predictable environments; as such, it promotes the preference for efforts directed at building the future, such as growing human and social capital. In contrast, a faster life history strategy evolved in harsh and unpredictable environments where it is optimal to focus on the present; as such, it is characterized by the preference for efforts directed at fulfilling immediate goals. Generally, fast life history strategists discount the future in favor of present gains (e.g., Wang et al., 2009; Griskevicius et al., 2011b). A faster life history strategy hedges against an uncertain future as individuals prioritize present survival and accelerated growth over investments for the future (Ellis et al., 2009).

Within this framework, procrastination, characterized by engaging in an activity that provides immediate hedonic rewards, is conceptualized as a manifestation of a fast life history strategy. As a fast life history strategy, procrastination may be the response to harsh and unpredictable environments where the likelihood of future success is low, hence serving the adaptive function of avoiding the cost of a current effort when there may not be a future in which the payoffs can be realized (Chen and Chang, 2016).

Existing data supports this proposition. Procrastination was positively associated with a fast life history orientation (Chen and Chang, 2016). Procrastination was higher when the environment was unpredictable and likelihood of future success was low (Chen and Qu, 2017; Chen and Kruger, 2017). Furthermore, the relation between environmental unpredictability and procrastination was mediated by life history strategy; environmental unpredictability was negatively related to a slower life history strategy, and in turn, slower life history strategy was negatively related to procrastination (Chen and Qu, 2017). Collectively, these findings suggest that an uncertain environment is likely to result in procrastination by psychologically shifting one’s resource allocation strategy to a faster one that favors present gains.

### The Present Research

Taking it all together, existing studies established that COVID-19 has resulted in a preponderance of procrastination among students in higher education (Aucejo et al., 2020; Biricik and Sivrikaya, 2020; Jia et al., 2020); yet, they do not elucidate how COVID-19 have effected an increase in procrastination. Drawing upon previous findings and employing a life history framework (Griskevicius et al., 2013; Del Giudice et al., 2015; Chen and Qu, 2017), we propose that the increase in procrastination may be attributed to the heightened levels of uncertainty in the pandemic. While it is not possible to compare the difference in uncertainty before and after the occurrence of the pandemic in this paper, we examine the change in uncertainty, and its effect on procrastination, across different phases of the pandemic. Specifically, we predict that students experience elevated levels of uncertainty, and correspondingly, higher levels of procrastination during the initial phase of the pandemic than subsequent phases.

For this paper, we examine the perceptions of students from a university based in Western Australia across two semesters. Following a declared state of emergency, social distancing restrictions began in Western Australia in early March 2020 (Government of Western Australia, 2020a). With the exception of essential services, public spaces were closed and gatherings were only limited to two people (GardaWorld, 2020a, b). Even though universities were exempt from the restrictions—as they fall under essential services, Western Australian universities prepared for closures of physical campuses and swiftly transited to online teaching and learning services (Pilat, 2020). At the university in which the sample of this paper was based in, these measures were implemented in the middle of a school semester. Classes were suspended for 2 weeks to allow for staff and students to prepare to transit to an online learning environment. Timetables had to be rescheduled to accommodate for such changes. However, classes that required the use of labs and clinics were under review. Collectively, these changes are likely to produce heightened levels of uncertainty among students during this school semester (February to May 2020).

COVID-19 restrictions started to ease in Western Australia by June 2020 (Government of Western Australia, 2020b). Social and recreational activities can resume with social distancing measures (limited number of people, two square meter per person capacity). Moreover, before the commencement of the following semester commenced, the university of this sample in which the paper was based on, announced that classes in the following semester were to be the same as the semester before. That is, classes and examinations were to take place online. Given student’s prior experience with online learning, and the improving COVID-19 situation, students are likely to experience less uncertainty in the following semester (August to November 2020) compared to the semester before.

Additionally, we further propose life history strategy as the psychological mechanism through which procrastination occurs: uncertainty in the pandemic climate psychologically shifts resource allocation strategies such that it is more optimal to favor present gains, which consequently results in procrastination. Specifically, we predict that uncertainty is associated with a faster life history strategy, which in turn, is positively related to procrastination. In doing so, we hope to shed light on the psychology behind the effect of uncertainty on procrastination, which is imperative for the design of procrastination interventions for the pandemic and “the new normal” post COVID-19.

## Materials and Methods

To examine the change in uncertainty, and its effect on procrastination, across different phases of the pandemic, data was collected across two time periods for this study. The first period (Time 1) was during the school semester in 2020, between August and November 2020 when COVID-19 surfaced and several changes were abruptly imposed, and the second period (Time 2) during the following school semester in 2021, between February and May 2021.

### Participants

A total of 253 participants were recruited through an Australian university’s subject pool system (201 females, *M*_age_ = 23.23, *SD*_age_ = 7.30). One hundred and forty six participants were recruited in Time 1 (118 females, *M*_age_ = 24.03, *SD*_age_ = 7.67) and 107 participants were recruited in Time 2 (83 females, *M*_age_ = 22.14, *SD*_age_ = 6.65). All participants were undergraduate students with the majority in their first year (*N*_Time 1_ = 52, *N*_Time 2_ = 62). Participants were only allowed to participate in this study once; participants who have responded to the survey in Time 1 did not participate in the survey conducted at Time 2.

### Procedure

After providing informed consent, participants responded to a series of questionnaires assessing uncertainty, life history strategy, and procrastination. For the survey in Time 1, participants were instructed to respond to the questions with reference to the period of time between March and May 2020 when COVID-19 cases spiked, and several measures were introduced in Western Australia and the university to curb the spread of COVID-19. For the survey in Time 2, participants were instructed to respond to the questions with reference to the period of time between August and November 2020 when COVID-19 was more managed. Participants provided demographic information before completing the study.

### Materials

#### Uncertainty

The Environmental Unpredictability Scale (Davis and Werre, 2008) was employed to assess participants’ perception of environmental uncertainty. Participants were asked to rate the chances they had of attaining future outcomes in three broad aspects: resource acquisition, offspring survivability, and social rank. Participants responded to items such as, “I will have a job that pays well,” “Life will turn out better for me than it has for my parents,” and “I will have a happy family life” for each aspect, respectively. Participants rated a total of 12 items on a 5-point scale (1 = *very low chance*, 5 = *very high chance*). The scores for each item was reversed coded and averaged to form a composite score for environmental uncertainty, where higher scores indicated higher perceived environmental uncertainty (*M* = 2.72, *SD* = 0.66, α_resource acquisition_ = 0.88, α_offspring survivability_ = 0.75, α_social rank_ = 0.73, α_overall_ = 0.88, ω = 0.75).

#### Life History Strategy

The life history strategy adopted by an individual was assessed with the Life History Strategy Short-Form scale (Figueredo et al., 2006). Participants responded to 20 statements (e.g., “I often make plans in advance” and “I avoid taking risks”) using a 7-point Likert scale (1 = “*strongly disagree*” to 7 = “*strongly agree*”). The items were reverse-scored and averaged to form a composite score; higher scores indicated the adoption of a faster life history strategy (*M* = 3.10, *SD* = 0.71, α = 0.77).

#### Procrastination

Procrastination on academic tasks was assessed by the Pure Procrastination Scale (Steel, 2010). Participants responded to 12 statements (e.g., “I delay making decision until it’s too late” and “Even after I make a decision I delay acting upon it”) on a 5-point scale (1 = *very seldom true of me*, 5 = *very often true of me*) (*M* = 3.21, *SD* = 0.83, α = 0.90).

## Analytical Strategy

Statistical analyses were performed using the software package SPSS Statistics for Windows, version 26.0 (IBM Corporation, 2019). Descriptive statistics were provided for environmental unpredictability, life history strategy, and procrastination. Before proceeding to the main analyses, the assumption of normality was checked. Values for skewness and kurtosis for all variables were between −1 and + 1, which were acceptable standards for a normal distribution (George and Mallery, 2010). This indicates that parametric tests can be employed in the subsequent analyses. Independent *t*-tests were conducted to compare the differences in means of environmental uncertainty and procrastination between two phases of the pandemic (i.e., Time 1 versus Time 2).

A multiple regression analysis was conducted to investigate if environmental uncertainty predicted procrastination. Gender, age, the school year in which participants were in,^1^ and the time at which the survey was conducted were also included in the regression model as control variables. Assumptions for regression analyses were also evaluated before the interpretation of its results. Inspection of the normal probability plot of standardized residuals and the scatterplot of standardized residuals against standardized predicted values indicated that the assumptions of normality, linearity, and homoscedasticity of residuals were met. Relatively high tolerances of all predictors in the regression model (Tolerance values were between 0.86 and 0.99) indicated that multicollinearity is not an issue. Examination of boxplots indicated the presence of 3 univariate outliers for environmental uncertainty. Mahalanobis distance exceeded the critical χ^2^ for df = 5 (at α = 0.01) of 15.09 for 6 cases in the data, indicating the presence of multivariate outliers. Multiple regression analyses were conducted with and without these univariate and multivariate outliers.

Finally, a mediation analysis using PROCESS version 3.1 (Hayes, 2018) was performed to examine if life history strategy mediated the relation between environmental uncertainty and procrastination. For mediation to be demonstrated, the bootstrap confidence interval of the indirect effect (path a^∗^b) must not include zero (bootstrap samples = 5,000) (Hayes, 2018). Similarly, mediation analyses were conducted with and without the univariate and multivariate outliers.

## Results

### Descriptive Analyses

Table 1 displays the means, standard deviations, skewness, and kurtosis of all the variables involved in this study. Table 2 displays the intercorrelations of the variables. Environmental uncertainty was negatively correlated with life history strategy and positively correlated with procrastination. Additionally, life history strategy was negatively correlated with procrastination.

**TABLE 1:** Descriptive statistics of all variables.

Variables	Time 1 (*N* = 146)				Time 2 (*N* = 107)			
	M	SD	Skew	Kurtosis	M	SD	Skew	Kurtosis
Environmental uncertainty	2.77	0.68	0.31	0.05	2.65	0.63	0.46	0.99
Life history strategy	3.00	0.68	0.60	–0.20	3.22	0.73	0.43	–0.39
Procrastination	3.21	0.82	–0.06	–0.88	3.19	0.85	–0.35	–0.15

**TABLE 2:** Intercorrelations of all variables.

Variables	1.	2.	3.
1. Environmental uncertainty	−		
2. Life history strategy	0.32**	−	
3. Procrastination	0.26**	0.21*	−

*p < 0.01;**p < 0.001.

### Main Analyses

An independent *t*-test was conducted to examine if environmental uncertainty was higher in Time 1 than in Time 2. Results indicated that environmental uncertainty was higher in Time 1 (*M* = 2.77, *SD* = 0.68) than in Time 2 (*M* = 2.65, *SD* = 0.63); however, this difference was not significant, *t*(251) = 1.40, *p* = 0.16, *d* = 0.18.

An independent samples *t*-test was conducted to examine if procrastination was higher in Time 1 than in Time 2. The analysis yielded no significant difference in procrastination between the two time periods, *t*(251) = 0.20, *p* = 0.84, *d* = 0.02. It is worth noting that procrastination was higher in Time 1 (*M* = 3.21, *SD* = 0.82) than in Time 2 (*M* = 3.19, *SD* = 0.85).

A multiple regression analysis was conducted to examine the effect of environmental uncertainty on procrastination. Results showed that the model accounted for a significant 9.2% of the variability in procrastination, *R*^2^ = 0.092, adjusted *R*^2^ = 0.074, *F*(5, 247) = 5.03, *p* < 0.01, *f^2^* = *0.10*. The analysis revealed that environmental uncertainty predicted procrastination, *B* = 0.32, *t*(247) = 4.15, *p* < 0.01, 95% CI [0.17, 0.47].^2^ Unstandardized (*B*) and standardized (β) regression coefficients for each predictor are reported in Table 3. A sensitivity analysis conducted using G-Power indicated that given a total sample size of 253, the minimum effect size to detect a power of 0.80 at α = 0.05 is *f^2^* = 0.04 for this study.

**TABLE 3:** Unstandardized (B) and standardized (β) regression coefficients for predictors in regression model predicting procrastination.

Variables		95% CI			
	*B*	LL	UL	se	β
Constant	2.34	1.65	3.03	0.35	
Environmental uncertainty	0.32***	0.17	0.47	0.08	0.26
Gender	0.12	–0.12	0.37	0.12	0.06
Age	–0.003	–0.02	0.01	0.01	–0.03
School year	0.23**	0.01	0.44	0.11	0.14
Time	–0.04	–0.25	0.16	0.11	–0.03

**p < 0.05; ***p < 0.01.

To examine if participants’ life history strategy mediated the relationship between perceived uncertainty and procrastination, a mediation analysis using Hayes’ PROCESS model 4 was conducted (Hayes, 2018). Environmental uncertainty was included as the independent variable, procrastination as the dependent variable, and life history strategy as the mediator. The time at which the survey was conducted was included as a covariate. Gender, age, and the school year participants were in were also included as covariates. Results revealed that environmental uncertainty was positively associated with life history strategy, *B* = 0.38, *p* < 0.01, 95% CI [0.26, 0.51], where higher perceived uncertainty predicted the adoption of faster life history strategies. Next, results also revealed that life history strategy was positively associated with procrastination, *B* = 0.17, *p* = 0.03, 95% CI [0.02, 0.32]. Participants with a faster life history strategy were more likely to procrastinate. Finally, results indicated that perceived uncertainty was positively associated with procrastination via participants’ life history strategy, *B* = 0.07, 95% *CI* = [0.003, 0.14], thus demonstrating the mediation effect of life history strategy on the relation between perceived uncertainty and procrastination.^3^ Unstandardized (*B*) regression coefficients, 95% confidence intervals, and *R*^2^ values for the mediation model are presented in Table 4.

**TABLE 4:** Mediation model coefficients for environmental uncertainty, life history strategy, gender, age, school year, time, and procrastination (*N* = 253).

Variables	*B*	LLCI	ULCI	se
DV = life history strategy (*R*^2^ = 0.18, *p* < 0.01)				
Constant	1.59	1.03	2.15	0.28
Environmental uncertainty	0.38***	0.26	0.51	0.06
Gender	0.34***	0.14	0.54	0.10
Age	0.002	–0.01	0.01	0.01
School year	–0.03	–0.20	0.14	0.09
Time	0.26***	0.09	0.43	0.09
DV = procrastination (*R*^2^ = 0.11, *p* < 0.01)				
Constant	2.07	1.35	2.79	0.37
Environmental uncertainty	0.25***	0.09	0.42	0.08
Life history strategy	0.17**	0.02	0.32	0.08
Gender	0.06	–0.19	0.31	0.13
Age	–0.004	–0.02	0.01	0.01
School year	0.23**	0.02	0.44	0.11
Time	–0.09	–0.30	0.12	0.11

**p < 0.05. ***p < 0.01.

## Discussion

This paper sought to elucidate how COVID-19 has effected an increase in procrastination. We proposed that the increase in procrastination may be attributed to the heightened levels of uncertainty in the pandemic. Moreover, we also examined the underlying psychological mechanism for *how* an uncertain climate drives procrastination. Specifically, we investigated life history strategy as the psychological mechanism through which procrastination occurs. Data was collected from undergraduate students across two time periods. Data collected at Time 1 assessed the perceptions of students in the semester where changes were abruptly imposed to stem the spread of COVID-19. Data collected at Time 2 assessed the perceptions of students one semester after changes to the curriculum were made. Our findings showed that environmental uncertainty and procrastination were similar between both semesters. We also found environmental uncertainty predicted procrastination. Furthermore, our results revealed that life history orientation mediated the relation between uncertainty and procrastination, suggesting that environmental uncertainty psychologically shifted the resource allocation strategies of students to a faster one such that it was more optimal to favor present gains, which consequently predicted procrastination.

Our findings showed that uncertainty was higher in Time 1 than in Time 2, even though this difference was not significant, it suggests that perceived uncertainty was especially heightened at the time when several changes were effected to curb the spread of COVID-19. This is consistent with anecdotal evidence that reported the uncertain futures students face relating to their education and professional careers (Aucejo et al., 2020; Jenei et al., 2020). The lack of significance in the difference in perceived uncertainty between the two time periods is likely due to the evolving nature of COVID-19 (e.g., new strain and sudden lockdowns due to new clusters of infected cases). As such, even though students may have accustomed to the changes made to their curriculum (e.g., online learning), which may lower uncertainty, being on a constant lookout for abrupt changes regarding the pandemic may keep uncertainty at relatively high levels for students. Moreover, individuals differ in their sensitivity toward uncertainty. Intolerance to uncertainty is the tendency to perceive and interpret uncertain situations as aversive and stressful (Dugas et al., 2004). Students’ intolerance to uncertainty may have influenced their perceptions of uncertainty during the pandemic, such that those with lower intolerance to uncertainty would have perceived less uncertainty than those with higher intolerance to uncertainty. Such individual difference could contribute to the similar levels of uncertainty perceived between the two time periods.

Our findings also demonstrated that procrastination was higher in Time 1 than in Time 2, though the difference in procrastination was not significant between the two time periods. While our results cannot conclude that procrastination levels were higher during the pandemic than before (Aucejo et al., 2020; Biricik and Sivrikaya, 2020; Jia et al., 2020), our finding showed that procrastination was higher during the initial phases of the pandemic than in the subsequent phases, suggesting that sudden changes brought about by COVID-19 played a part in encouraging procrastination. The lack of significant difference in procrastination may be an artifact of the similarity in environmental uncertainty levels. Additionally, as procrastination is often engaged to cope with negative emotional states (Hen and Goroshit, 2014), and elevated negative emotional states were also constantly reported during the pandemic (Biricik and Sivrikaya, 2020; Jenei et al., 2020; Rahimi and Vallerand, 2021), it is likely the affective state of participants contributed to similar levels of procrastination observed between the time two periods.

We also found that uncertainty predicted procrastination, and that life history orientation mediated the relation between uncertainty and procrastination, which is consistent with the life history framework (Chen and Qu, 2017; Chen and Kruger, 2017). In line with this theoretical framework, our results demonstrated that environmental uncertainty predicted a faster life history strategy, which signaled that it was more optimal to favor present gains than future ones, consequently predicting procrastination. Our findings also provided further support to the conceptualization of procrastination as an adaptive response to environmental conditions—specifically, to environmental unpredictability (Chen and Qu, 2017; Chen and Kruger, 2017). Moreover, consistent with previous findings, our findings also showed that procrastination was associated with a faster life strategy, suggesting that procrastination is more likely when present gains are favored (Chen and Chang, 2016; Chen and Qu, 2017). This also corroborates findings that demonstrated the association procrastination and negative attitudes of academic investment have with reductions in future time perspective and future outlook (Ferrari and Díaz-Morales, 2007; Schechter and Francis, 2010).

Although we found life history strategy to mediate the association between uncertainty and procrastination, the mediation effect is relatively small. This suggests that there could be factors that influenced the mediating effect of life history strategy—one factor could be an individual’s childhood socioeconomic status. Within life history theory, an individual’s early life environment determines the life history strategy they adopt (Del Giudice, 2009; Ellis et al., 2009; Griskevicius et al., 2011a). Individuals who grew up in low socioeconomic environments were more likely to be exposed to environmental stressors, such as fluctuating resource availability and changing household memberships, which prompts them to adopt faster life history strategies compared to individuals who grew up in high socioeconomic environments (where environmental stressors were largely absent) (Belsky, 2007). This early exposure to environmental stressors not only affects the life history strategy one adopts during their childhood, it also affects how individuals respond to environmental stressors later in life (Caretta et al., 1995). Individuals who have developed a faster life history strategy tendency as a function of their early life low socioeconomic environments are sensitive to environmental stressors and likely to discount the future in favor of present gains (Boyce and Ellis, 2005; Griskevicius et al., 2011b). As such, it is likely that these individuals will be more sensitive to the uncertain climate during the pandemic and hence, are more responsive in adopting a faster life history strategy than individuals who grew up in high socioeconomic environments (and have developed a tendency to adopt slower life history strategies). This difference in response to environmental uncertainty could explain the small mediation effect observed.

### Limitations and Future Directions

Our work is far from conclusive and poses questions for future work. As we have discussed previously, childhood socioeconomic status can potentially influence one’s sensitivity to environment stresses and shifts in life history strategy. Given that we only measured participants’ life history strategy based on their current environment than their early life environment, and childhood socioeconomic status was not assessed in this study, we are not able to ascertain the extent to which participants’ childhood socioeconomic status affected participants’ reaction to heightened levels of uncertainty during the pandemic. Future studies should assess participants’ childhood socioeconomic status and test for a moderated mediation model, where life history strategy mediates the relation between uncertainty and procrastination and childhood socioeconomic status moderates the shift in life history orientation in response to environmental uncertainty.

Additionally, we did not account for personality traits that may influence one’s tendency to procrastinate. The tendency to procrastinate has also been associated with personality traits, specifically, conscientiousness and neuroticism. Conscientiousness was inversely correlated to procrastination (Johnson and Bloom, 1995; Van Eerde, 2003; Steel, 2007). Neuroticism was also significantly correlated to procrastination (Johnson and Bloom, 1995). With these personality traits predicting procrastination, it would be difficult to tease apart the unique effects of environmental uncertainty from the effects of these traits on procrastination.

We recognize that our study is limited to self-reported measures, which may limit interpretation of our findings, especially since the internal consistency of the measures assessing environmental uncertainty (α _social rank_ = 0.73) and life history strategy (α = 0.77) were relatively lower, though still within acceptable range (Cortina, 1993), than the rest. Given that uncertainty have been associated with physiological changes, such as heart rate (Averill et al., 1972; Monat et al., 1972), future studies can consider measuring for physiological changes on top of self-reported perceived environmental uncertainty. Life history strategy can also be assessed behaviorally by observing how they interact with others—fast life history strategists tend to express criticism and talk with physical animations (Sherman et al., 2013). Hence, future studies can consider employing these other means of assessment to complement the self-reported measures of environmental uncertainty and life history strategy.

Furthermore, as participants often evaluate their behaviors negatively when asked to think about them retrospectively, self-reported measures of procrastination may not accurately reflect actual procrastination (Steel et al., 2001; Moon and Illingworth, 2005). Thus, it is likely that procrastination scores were inflated—as an artifact of using a self-report measure—and not truly reflective of participants’ actual procrastination in this study. To overcome this shortcoming, future studies should consider employing observed measures for assessing actual procrastination.

With the adverse effect procrastination has on academic performance (Kim and Seo, 2015), it is important that procrastination is managed. Given that an emphasis on present gains predicts procrastination, one way to ameliorate procrastination in students would be to shift the emphasis to the future by boosting their perceived value of the future. This can be done by making the future self a salient concept in students as students have found that a future self can motivate action. An event-related fMRI study found that future self-continuity reduced temporal distancing—the extent to which individuals distinguish between the present self and future self; when individuals perceived their future-self more clearly, they made better decisions for their future (Ersner-Hershfield et al., 2009). Increasing the congruence between the present and future self was found to generate motivation for current action (Lewis and Oyserman, 2015). Self-focused mental imagery can be used as a psychological tool to bridge the present-future gap to reduce procrastination (Blouin-Hudon and Pychyl, 2017). As an uncertain climate is likely to persist, eliminating procrastination by reducing uncertainty may not necessarily be an ineffective strategy. Having identified life history orientation as the psychological mechanism behind uncertainty and procrastination, future studies can examine the feasibility in shifting students’ emphasis on the future to minimize procrastination.

## Conclusion

This paper demonstrated that levels of uncertainty and procrastination in undergraduate students were similar between the semester where COVID-19 changes are abruptly imposed to stem the spread of COVID-19 and the following semester. Employing an evolutionary life history framework, this paper found that uncertainty predicted procrastination. This paper also provided an underlying explanation for *how* an uncertain climate drives procrastination. Specifically, the findings of this paper showed that uncertainty in the current pandemic prompted students to psychologically shift their life history strategy such that it was more optimal to focus on present gains, which consequently predicted procrastination.

## Data Availability Statement

The raw data supporting the conclusions of this article will be made available by the authors, without undue reservation.

## Ethics Statement

The present study has been approved by the Murdoch University Research Ethics with the following approval reference number: 2020/145. The patients/participants provided their written informed consent to participate in this study.

## Author Contributions

AL and SJ were involved in the conceptualization of the project and formulation the study design. AL acquired and analyzed the data and drafted the manuscript. Both authors read and approved the final manuscript, agreed to be accountable for the content of this manuscript.

## Conflict of Interest

The authors declare that the research was conducted in the absence of any commercial or financial relationships that could be construed as a potential conflict of interest.

## Publisher’s Note

All claims expressed in this article are solely those of the authors and do not necessarily represent those of their affiliated organizations, or those of the publisher, the editors and the reviewers. Any product that may be evaluated in this article, or claim that may be made by its manufacturer, is not guaranteed or endorsed by the publisher.

## Footnotes

1 The school year participants were in were coded as 1 = first years and 0 = non-first-years.

2 A similar regression analysis was conducted with the exclusion of univariate and multivariate outliers. The analysis excluding the outliers revealed similar finding. The model accounted for a significant 9.3% of the variability in procrastination, *R*^2^ = 0.093, adjusted *R*^2^ = 0.074, *F*(5, 239) = 4.89, *p* < 0.01. The analysis revealed that environmental uncertainty predicted procrastination, *B* = 0.33, *t*(239) = 3.99, *p* < 0.01, 95% CI [0.17, 0.49]. The significance of the effect of uncertainty on procrastination remained.

3 A similar mediation analysis was conducted with the exclusion of univariate and multivariate outliers. The analysis excluding the outliers revealed similar findings: Eenvironmental uncertainty was positively associated with life history strategy, *B* = 0.37, *p* < 0.01, 95% CI [0.22, 0.52]. Life history strategy was positively associated with procrastination, *B* = 0.19, *p* = 0.02, 95% CI [0.03, 0.35]. Perceived uncertainty was positively associated with procrastination via participants’ life history strategy, *B* = 0.07, 95% *CI* = [0.006, 0.14], indicating that life history strategy mediated the relation between environmental uncertainty and procrastination.
